# Food-Grade Nanoemulsions: Preparation, Stability and Application in Encapsulation of Bioactive Compounds

**DOI:** 10.3390/molecules24234242

**Published:** 2019-11-21

**Authors:** Qingqing Liu, He Huang, Honghong Chen, Junfan Lin, Qin Wang

**Affiliations:** 1 Key Laboratory of Grain and Oil Processing and Food Safety of Sichuan Province, College of Food and Bioengineering, Xihua University, Chengdu 610039, China; liuqing_861006@163.com (Q.L.);; 2 Department of Nutrition and Food Science, College of Agriculture and Natural Resources, University of Maryland, College Park, MD 20740, USA

**Keywords:** nanoemulsions, preparation, stability, application, encapsulation

## Abstract

Nanoemulsions have attracted significant attention in food fields and can increase the functionality of the bioactive compounds contained within them. In this paper, the preparation methods, including low-energy and high-energy methods, were first reviewed. Second, the physical and chemical destabilization mechanisms of nanoemulsions, such as gravitational separation (creaming or sedimentation), flocculation, coalescence, Ostwald ripening, lipid oxidation and so on, were reviewed. Then, the impact of different stabilizers, including emulsifiers, weighting agents, texture modifiers (thickening agents and gelling agents), ripening inhibitors, antioxidants and chelating agents, on the physicochemical stability of nanoemulsions were discussed. Finally, the applications of nanoemulsions for the delivery of functional ingredients, including bioactive lipids, essential oil, flavor compounds, vitamins, phenolic compounds and carotenoids, were summarized. This review can provide some reference for the selection of preparation methods and stabilizers that will improve performance in nanoemulsion-based products and expand their usage.

## 1. Introduction

Nanoemulsions, exhibiting droplet sizes of <200 nm, represent liquid-in-liquid dispersions that are kinetically stable. Water and oil are the two incompatible liquids most extensively applied in commercial environments. Because of their small size, characteristics such as visible transparency, high surface area per unit volume, sound stability and tunable rheology are often observed. Additionally, large-scale nanoemulsions’ preparation is easily achievable in industrial conditions. Therefore, nanoemulsions are especially suitable for commercial applications [1,2,3]. 

Since the oil and water phases are distributed relatively spatially, simple nanoemulsions can be divided into oil-in-water (O/W) nanoemulsions denoting the dispersion of small oil droplets in an aqueous medium, and water-in-oil (W/O) nanoemulsions signifying small water droplets distributed in an oil medium [3]. Additionally, utilizing a two-step procedure, it is also possible to produce two types of multiple nanoemulsions, namely water-in-oil-in-water (W/O/W) or oil-in-water-in-oil (O/W/O) [4]. For instance, the preparation of W/O/W nanoemulsions is achieved by assimilating the oil phase comprising lipophilic surfactant with the water phase to form the initial W_1_/O nanoemulsions, which are then homogenized with an additional water phase (W_2_) comprising hydrophilic surfactant [5]. 

The methods used for nanoemulsions’ preparation can be divided into two principal groups namely low-energy and high-energy techniques. When environmental factors (e.g., composition or temperature) or nanoemulsions’ compositions are modified, small droplets are generated, providing the basis necessary for the successful operation of the low-energy methods [3,6,7,8]. High-energy methods usually consume significant energy (~10^8^–10^10^ W/kg) to form small droplets. Furthermore, in the utilization of high-energy methods, the oil and water phases are breached and blended using the powerful cavitational, shear and turbulent flow profiles created by the specifically designed devices [9,10].

Nanoemulsions are thermo-dynamically unstable since the free energy required to separate the oil phases from the water phases is lower than what is necessary for emulsification. Therefore, nanoemulsions typically break down during storage due to various mechanisms, such as gravitational separation (creaming or sedimentation), flocculation, coalescence and Ostwald ripening [11]. Moreover, various chemical and biochemical reactions such as flavor loss, biopolymer hydrolysis, color fading and lipid oxidation can adversely affect nanoemulsions, causing them to degrade during storage or lose their acceptable quality characteristics. Among the chemical deterioration phenomena mentioned above, lipid oxidation occurs the most frequently in nanoemulsions [12]. 

For several commercial uses, it is crucial that nanoemulsions-based products remain physiochemically stable when exposed to unfavorable environmental conditions (including temperature, mechanical forces, and ionic strength) during their production, storage, transportation and application [3,6]. The addition of suitable stabilizers, including emulsifiers, weighting agents, texture modifiers and ripening inhibitors can improve the physical stability of nanoemulsions [6,13]. Given that, three methods are commonly used to improve the nanoemulsions’ chemical stability, including the manipulation of interfacial characteristics (e.g., thickness, charge, and chemical reactivity), the addition of chelating agents or antioxidants, as well as controlling environmental elements (e.g., temperature, light, pH, and oxygen levels) [3,6].

So far, a number of food ingredients and additives, including bioactive lipids, vitamins, flavorings, acidulants, preservatives, colorings, antioxidants and so on, have been encapsulated by nanoemulsions and some of them are already available in the market [1,3,14]. A larger droplet surface area, as well as a decline in particle size of the nanoemulsions may lead to increased functionality of the bioactive compounds contained within them. The majority of the bioactive compounds are characteristically lipophilic. Thus, O/W nanoemulsions are commonly used to improve the solubility and dispersibility of lipophilic substance in aqueous media, enhance stability, appearance, taste or texture, increase uptake absorption and bioavailability, and reduce the off-flavor (such as bitterness or astringency) [14,15,16]. 

In this review article, we will focus on the simple nanoemulsions and discuss the most relevant research from the literatures in the last five years on nanoemulsions’ fabrication, stabilization and application. This review paper contains five sections. The first section is the introduction. The second section summarizes the various methods to prepare nanoemulsions. In the third section, we discuss the physically destabilization mechanisms of nanoemulsions such as gravitational separation (creaming or sedimentation), flocculation, coalescence and Ostwald ripening. Moreover, the chemical stability of nanoemulsions are also discussed in this section. The fourth section reviews the stabilizers used in the nanoemulsions, such as emulsifiers, texture modifiers, weighting agents, ripening inhibitors and other components. The fifth section discusses the application of nanoemulsions in encapsulation of bioactive compounds in the last five years.

## 2. Preparation

As described in Section 1, a number of methods were developed to facilitate nanoemulsions, which include high-energy as well as low-energy techniques [17]. Selecting an appropriate method for the preparation of nanoemulsions rely on the characteristics of the compounds needing homogenization (specifically the surfactant and oil phases), as well as the required physicochemical attributes and operational qualities of the ultimate product (including rheological, optical, release, and stability properties) [6]. Understanding the various fabrication methods is crucial for relevant personnel to choose the most suitable preparation technique and fabricate nanoemulsions for special application. 

### 2.1. Low-Energy Methods

**Figure 1:**
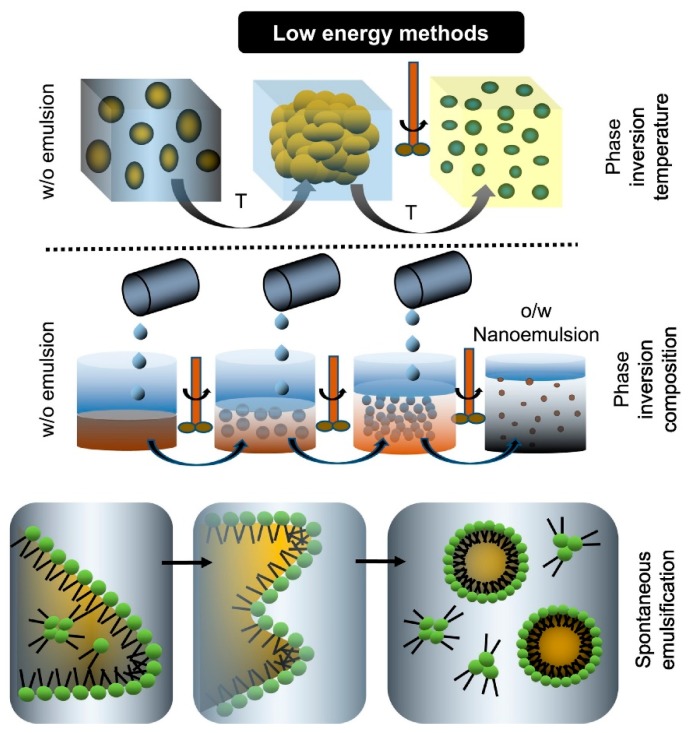
Schematic depiction of the characteristic low-energy techniques used to create O/W nanoemulsions, including phase inversion temperature (PIT), phase inversion composition (PIC) and spontaneous emulsion (SE) [19].

Low-energy methods are denoted by changes in environmental conditions, as well as the composition of the mixture influencing the development of oil nanodroplets within the mixed systems containing surfactants, oil, and water. The most frequently used low-energy techniques are spontaneous emulsion (SE), emulsion phase inversion (EPI) (including phase inversion composition (PIC), and phase inversion temperature (PIT)) [8,18]. The principles of the characteristic low-energy techniques used to O/W nanoemulsions were shown in Figure 1.

In this section, the low-energy methods for nanoemulsions’ preparation and their application in encapsulation of bioactive compounds are summarized in Table 1 and introduced as follows.

#### 2.1.1. Spontaneous Emulsion (SE)

SE, also named as emulsification by solvent diffusion (ESD), can take place through numerous mechanisms [10] and provides the potential for an affordable approach. The spontaneous formation of an O/W nanoemulsion facilitated by specific temperatures relies on the chemical compounds present in both phases, as well as the utilized emulsifier [34]. Whether surfactants are present or not, the utilization of solvents can facilitate this spontaneous process [35,36]. From the points of view of cost, flavor, and safety, the use of solvents is usually problematic in the food industry. Therefore, the SE process usually includes adding an organic phase consisting of a hydrophilic surfactant and oil into an aqueous phase consisting of water and potentially a co-surfactant [37]. 

Kinetic barriers can cause SE to occur slowly, while the ouzo effect initiates this process immediately [10]. The ouzo effect results from a significant supersaturation of the oil, facilitating the nucleation of oil droplets when combined with water. Consequently, instantaneous diffusion of the oil to the closest droplet occurs and decreases the supersaturation to avoid any further nucleation [36]. 

Barzegar et al. prepared nanoemulsions by SE and found that the best nanoemulsions with droplet size of around 50 nm were formed [20]. Zhao et al. prepared three types of essential oil nanoemulsions by SE. At the same surfactant level, the nanoemulsions containing different essential oil displayed particle sizes of about 10–30 nm, 10–30 nm and 50–500 nm, respectively [21]. Yildirim et al. prepared stable nanoemulsions by SE. The best nanoemulsions with particle size of ~100 nm exhibited high physical stability and antimicrobial activity [22]. Tian et al. prepared nanoemulsions by SE. Stable nanoemulsions were obtained [23]. Ghiasi et al. prepared nanoemulsions by SE. The average droplet diameter in the most superior nanoemulsions were 13–14 nm, while its continued stability exceeded a storage period of eight months in extreme temperatures such as 4 °C and 45 °C [24]. Using SE, Wang et al. produced nanoemulsions displaying a clearly established diameter of approximately 109−139 nm, a negative surface zeta-potential ranging between −28.5 mV and −35.8 mV, as well as a spherical structure [25]. 

#### 2.1.2. Emulsion Phase Inversion (EPI)

EPI, also known as catastrophic phase inversion (CPI), usually refer to the creation of W/O nanoemulsions using traditional high-speed mixers. Then, these W/O nanoemulsions are transformed into O/W nanoemulsions by modifying the temperature or the composition, that is PTC and PIC methods [10]. Specifically, the PIC and PIT approaches denote transitional inversion resulting from changing factors (temperature or composition), which influence the hydrophilic lipophilic balance (HLB) in the system [38].

##### Phase Inversion Composition (PIC)

PIC, also named emulsion inversion point (EIP) [39], involves procuring O/W nanoemulsions from their W/O analogs via a shift in the natural emulsifier curvature by altering the volume fraction of the water at a given temperature. The nano-emulsification process using PIC involves the gradual addition of the water phase to the oil phase, resulting in the steady increase of the water volume fraction. A phase inversion can take place at a specific level, leading to the emergence of a bicontinuous phase, which can ultimately capture oil phases into water phase and form O/W nanoemulsions [40].

The PIC method has many advantages, including low cost and the need for simple apparatus. However, the preparation time of this process was longer than that of SE technique due to the smaller driving forces of PIC method.

Zhang et al. prepared stable nanoemulsions by using the EIP method. The particle sizes of the prepared nanoemulsions were <200 nm [26]. Borrin et al. produced nanoemulsions by EIP method and incorporated them in pineapple ice creams to replace artificial yellow dyes [27,28]. Nantarat et al. prepared nanoemusions by PIC. The average droplet size of the nanoemulsions produced was between 29.55 to 37.12 nm [29]. 

##### Phase Inversion Temperature (PIT)

Nano-emulsification by PIT is also premised on transitional inversion, which is prompted by HLB variations in the system, resulting from fluctuations in temperature [2]. Temperature-sensitive surfactants become water-soluble a low temperature, while a positive surfactant layer curvature is evident at the droplet interface. By contrast, the surfactants become oil-soluble at a high temperature, with a negative surfactant layer curvature apparent at the droplet interface [41]. An intermediate temperature (PIT) causes the surfactants to exhibit a similar affinity to both the oil and water phases, consequently, producing a zero value for the spontaneous surfactant layer curvature at the droplet interface [42]. Therefore, the oil in a lamellar liquid crystalline phase or bicontinuous phase is completely solubilized [43]. The advantages of the PIT method are that it is a low-energy approach without requiring high shear forces [44]. 

Chuesiang et al. fabricated nanoemulsions using the PIT method, where a mixture of oil, water and surfactant was heated above the PIT and then quench cooled by stirring. Nanoemulsions with particle size of 101 nm were obtained [30,31]. Su et al. fabricated nanoemulsions by the PIT method. The best nanoemulsions had a particle size of approximately 100 nm and retained stability after a 15-day storage period [32]. Jintapatanakit et al. prepared nanoemulsions by the PIT method. The droplets size of nanoemulsions were 20–100 nm and polydispersity index (PDI) was below 0.2 [33]. 

### 2.2. High-Energy Methods

**Figure 2:**
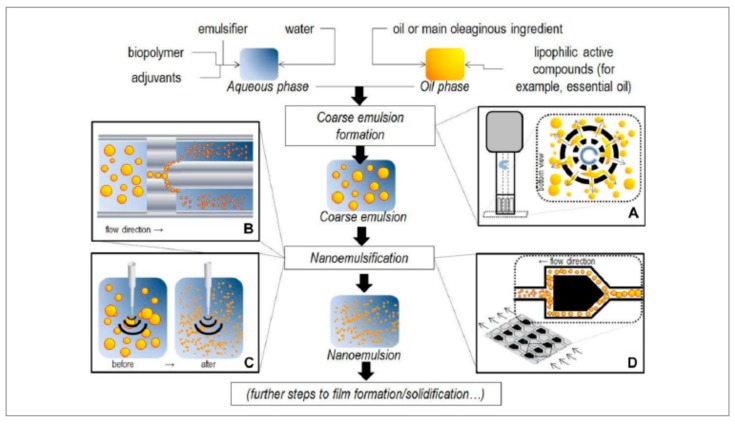
Schematic portrayal of high-energy techniques utilized for the preparation of O/W nanoemulsions. (A) traditional high-speed mixers are usually employed to form a coarse O/W emulsions before emulsification by (B) high-pressure homogenization (HPH), (C) ultrasonic homogenization (USH), (D) high-pressure microfluidic homogenization (HPMH) [10].

High-energy methods, denote mechanical techniques, employing mechanical equipment to separate the dispersed phase into droplets inside the continuous phase to generate forces that are highly disruptive [10]. Since high-energy methods permit the utilization of non-toxic/natural emulsifiers at lower concentration levels, they are more appropriate for food-related nanoemulsions preparation, while they are also expedient for production at an industrial scale and the necessary equipment is available commercially [14]. Usually, two steps are involved when producing O/W nanoemulsions using high-energy methods. Firstly, the coarse O/W emulsions were formed by mixing of the components using a high-speed mixer or stirrer. Later on, the coarse emulsions are exposed to disruptive forces to facilitate a reduction in the droplet diameter to 200~500 nm [10]. Based on the devices used, high-energy methods include rotor-stator emulsification (RSE), high-pressure homogenization (HPH), high-pressure microfluidic homogenization (HPMH) and ultrasonic homogenization (USH) [9,45]. The principles of high-energy techniques used to create O/W nanoemulsions were shown in Figure 2.

In this section, the high-energy methods for nanoemulsions’ preparation and their application in encapsulation of bioactive compounds are summarized in Table 2 and introduced as follows.

#### 2.2.1. Rotor-Stator Emulsification (RSE)

RSE is also known as high-speed homogenization (HSH). Various industries including food, cosmetics and pharmaceutical companies commonly use rotor-stator mixers to produce emulsions. They are usually considered as a standard method for emulsion preparation regarding dispersed phase volume fractions and intermediate-to-high viscosity [56,57].

The hydrodynamic intensity and the size of the subsequent emulsion droplets depend on the speed of the rotor, which commonly ranges 10 m/s and 30 m/s in industrial applications. The decrease in mean droplet size is fairly low after migrating through the rotor-stator region. Therefore, several migrations were necessary to achieve a steady-state droplet size, particularly for the formation of the small droplet sizes essential to nanoemulsions [9]. However, the exclusive use of RSE is difficult to produce nanoemulsions.

Karthik et al. prepared nanoemulsions by three methods (HSH, HPH and HSH + HPH). Among them, the particle size of nanoemulsion obtained by HSH was 87 nm [46]. 

#### 2.2.2. High-Pressure Homogenization (HPH)

HPH is a commonly used method in industrial nanoemulsion preparation [58]. It is used to decrease the droplet size of coarse pre-emulsions (often prepared with a rotor-stator mixer) into nano-size with narrow distribution [9]. HPH devices function on the same principle as those employed for the amalgamation of beverages. A piston pump is used to push the macroemulsions through a narrow valve situated downstream. During this procedure, the extreme hydraulic shear and turbulence breaks apart the macroscale droplets to form smaller ones, and is repeated several times until the successful formation of nanoemulsions [10,59]. Three main variables can be a matter of optimization for HPH: number of pass cycles, working pressure, and system temperature [60].

Galvão et al. prepared nanoemulsions by using HSH (Ultra-Turrax), followed by HPH. The average droplet size of nanoemulsions was 132 ± 2.0-145 ± 1.0 nm, which depended on the number of cycles and working pressure applied [47]. Ma et al. prepared nanoemulsions using the HPH method. The particle size of nanoemulsions was 203.6–260.6 nm [48]. Dey et al. produced nanoemulsions via the use of HPH. The particle size, PDI and zeta-potential of nanoemulsions obtained were 89.7 ± 27.7 nm, 0.226 ± 0.021 and −12.54 ± 1.67 mV, respectively [49].

#### 2.2.3. High-Pressure Microfluidic Homogenization (HPMH)

Microfluidic devices were used successfully in the development of nanoemulsions [14]. Indeed, using HPMH can facilitate the large-scale production of size-tailored nanoemulsions. It has been stated that HPMH is more effective than HPH during the formation of nanoemulsions subjected to a single pass at an equal working pressure [10,60]. The HPH method and HPMH method are inherently similar. Both employ a high-pressure positive displacement pump to create nanoemulsions, generally at a setting between 30 MPa and 120 MPa, but the specific device design in each case differ considerably [10].

Llinares et al. prepared nanoemulsions by microfluidization. The best nanoemulsions showed the lowest mean particle size (2.88 nm) [50]. According to Karthik et al., O/W nanoemulsions were prepared by microfluidization with different emulsifiers. The nanoemulsions showed the lowest mean particle size of 148 nm [51]. Raviadaran et al. produced and optimized nanoemulsions via the use of a microfluidizer. Stable nanoemulsions with particle size of 275.5 nm, PDI of 0.257, zeta-potential of −36.2 mV, and viscosity of 446 cP were obtained [52]. As reported by Liu et al., nanoemulsions were prepared using a dual-channel high-pressure microfluidizer. Stable nanoemulsions were obtained with particle size of <160 nm [53].

#### 2.2.4. Ultrasonic Homogenization (USH)

Nanoemulsions can be effectively formed using USH devices. Inserting a sonication probe into the prepared coarse emulsion promotes the generation of mechanical ultrasound vibrations, which induces the formation and collapse of microbubbles in close proximity of the sonication probe. Therefore, hotspots, high shear forces, and turbulence are created, ultimately resulting in effective nanoemulsions’ droplet disruption toward the nanoscale [61]. The nanoemulsions’ morphology is affected by power, frequency/amplitude of the ultrasound waves, and treatment time. Additionally, hydrostatic pressure, dissolved gas concentration, apparatus configuration, and temperature are also important in the nano-emulsification processes performed using USH devices [62]. 

Laboratory-scale ultrasonic homogenizer has many benefits, such as simple operation, excellent energy efficiency, and affordability. Additionally, there are more potential advantages at optimized conditions, such as low emulsifier content requirements, excellent dispersion stability, and a decreased risk of microbial contaminants entering the processing stage [62]. However, fabricating ultrasonic devices suitable for industrial application remains challenging. Furhtermore, additional defects exist in using sonication to prepare nanoemulsions. These include the emergence of hotspots, as well as deterioration processes prompted by cavitation that may prove damaging to mutable components [14]. Moreover, abrasion of the sonication probe induced by cavitation increases the risk or releasing metal ions into the emulsion [63].

Kumar et al. prepared nanoemulsion by the USH method. The average sizes of two different nanoemulsions were 24.48 ± 5.70 nm and 20.41 ± 3.41 nm, respectively [54]. Moghimi et al. prepared nanoemulsion by the USH method. The most stable nanoemulsions were produced with the particle size of 171.88 ± 1.57 nm [55].

#### 2.2.5. Combined Methods

Recently, more and more nanoemulsions were prepared by combined methods in order to overcome the drawbacks of a single method. 

Karthik et al. prepared nanoemulsions by HSH, HPH and HSH + HPH. The results showed that HPH involved emulsification process (HPH and HSH + HPH) produced nanoemulsions that display stability regarding morphology, particle size, and other physical attributes [46].

## 3. Stability

### 3.1. Physical Stability

**Figure 3:**
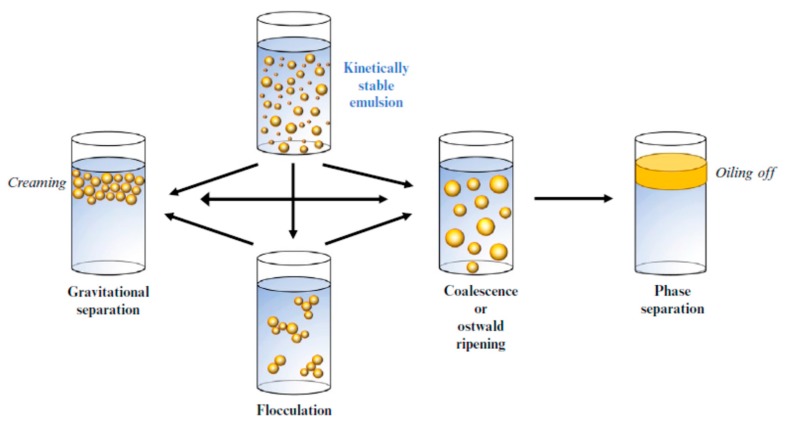
Schematic representation of the mechanisms responsible for nanoemulsion physical instability (phase separation): gravitational separation, flocculation, coalescence and Ostwald ripening.

The undesirable molecular interactions at the oil-water interface as a result of the hydrophobic effect induces thermodynamic instability in nanoemulsions [59]. Nanoemulsions will eventually degrade as a result of several mechanisms such as flocculation, gravitational separation, coalescence, phase separation, and Ostwald ripening, as shown in Figure 3 [3,6]. Understanding the essential mechanisms responsible for nanoemulsions’ instability is crucial in developing systems exhibiting adequate stability qualities.

#### 3.1.1. Gravitational Separation

The process in which nanoemulsion droplets move downward (sedimentation) or upward (creaming) due to their density exceeding or being lower than that of the liquid surrounding it, is known as gravitational separation. Water tends to move in a downward direction, while oil migrates upward since most liquid oils are less dense than water in a liquid state. Therefore, sedimentation is common in W/O nanoemulsions, while O/W exhibit more cases of creaming [3,6]. An increase in droplet size, density contrast in conjunction with a decline in the aqueous phase viscosity, influence the motion speed of the droplets induced by gravitational separation [6]. 

According to Arancibia et al., the inferior stability of nanoemulsions containing 15% avocado oil and 8% starch could be ascribed to gravitational separation of phases caused by fat droplet flocculation/coalescence and starch precipitation/aggregation [64]. According to Chen et al., due to gravitational separation (sedimentation), pure cinnamaldehyde could not produce stable nano-emulsions. The oil droplets moved downwards in the pure cinnamaldehyde system due to the higher density of cinnamaldehyde (1050 kg m^-3^) than that of room temperature water (997 kg m^-3^) [65]. 

#### 3.1.2. Flocculation and Coalescence

The colloidal interaction between the droplets determines two types of droplet accumulation namely coalescence and flocculation [6]. 

The process by which two or more droplets attract each other to form clusters is known as flocculation [6]. As reported by Bai et al., utilizing amphiphilic polysaccharides above a certain level as emulsifiers could promote emulsion instability, which was attributed to exhausted flocculation mechanism, could diminish the stability of some nanoemulsion products in the long run [66]. As reported by Li et al., D-limonene nanoemulsions became unstable and tended to flocculate and coalesce, which caused a variation in zeta-potential at the storage temperatures [67]. As reported by Bai et al., a cream layer was evident for saponin-coated droplets when salt concentrations exceeded 300 mM, suggesting that the accumulation of droplets primarily resulted from flocculation instead of coalescence [68].

The process by which a larger droplet is formed when several droplets collide and amalgamate is known as coalescence [6]. As reported by Bai et al., rhamnolipids were able to stabilize O/W nanoemulsions. The droplets coated with rhamnolipids displayed stability following thermal treatments between 30 °C to 90 °C, with salt concentrations below 100 mM NaCl, pH levels between 5 and 9, as well as storage for at least two weeks at room temperature. However, nanoemulsions were unstable when subjected to storage conditions that were extremely acidic (pH 2–4) or in the presence of high ionic strength (200–500 mM NaCl). These results were ascribed to coalescence caused by a decline in the electrostatic repulsion among the droplets in the range and magnitude at high salt and low pH levels [68]. Wooster et al. prepared triglyceride nanoemulsions by the HPMH method, using a combination of Span 80 and Tween 80 as emulsifiers and n-alcohol as a co-solvent. However, addition of an excess of n-alcohol led to nanoemulsion destabilization, coalescence was found to be the primary destabilization mechanism [69]. According to Shu et al., nanoemulsions containing astaxanthin stabilized by saponins displayed an extremely high sensitivity and were predisposed to droplet coalescence when high salt concentrations were present. These results could be ascribed to a decline in the electrostatic repulsion among the negatively charged droplets facilitated by the presence of Na^+^ cations, and inducing the coalescence and instability of the droplets [70].

#### 3.1.3. Ostwald Ripening

Driven by the curvature differences of the particles, dispersed phase molecules diffuse through the continuous phase causing the expansion of larger droplets and the shrinkage of smaller droplets in a process known as Ostwald ripening, which is the primary instability mechanism for such nanoemulsions. Ostwald ripening occurs when the dispersed phase solubility in large droplets (small curvature) is lower than in small droplets (large curvature), which prompts droplet growth due to the appearance of a concentration gradient [71].

According to Ryu et al., nanoemulsions containing essential oil are especially susceptible to Ostwald ripening because of a significant oil phase solubility in the aqueous phase even though its predominantly hydrophobic nature [72]. According to Walker et al., during or soon after homogenization, nanoemulsions containing thyme oil experienced swift droplet growth when low levels of fish oil (<75%) were present. The reason was that the relatively high water-solubility of thyme oil induced expeditious droplet growth as a result of Ostwald ripening [73].

### 3.2. Chemical Stability

Various biochemical and chemical reactions such as flavor loss, lipid oxidation, biopolymer hydrolysis and color fading occur in nanoemulsions leading them to lose their favorable characteristics. Of these, lipid oxidation is considered as one of the most significant types of chemical degradation [12]. The interfacial areas of nanoemulsions are relatively large, leading to accelerated lipid oxidation due to water-soluble pro-oxidants (e.g., transition metals) coming into contact with oil-soluble reactants (e.g., polyunsaturated lipids and hydroperoxides) [74]. Park et al. suggested that the long-term stability of nanoemulsions might exhibit a more significant association with the chemical stability instead of the physical stability in the case of O/W nanoemulsions with retinol [75].

### 3.3. Correlative Instability Mechanism

Each instability mechanism is always associated with others or they appear simultaneously [8]. Powell et al. prepared nanoemulsions using Pluronic F68 and various oils generally utilized for pharmaceutical and cosmetic applications, and explored their stability mechanisms. The eventual destabilization appeared due to the rising of large drops which formed through coalescence and Ostwald ripening and coalescence were responsible for the formation of large drops, which rose to cause the ultimate destabilization [76]. According to Chen et al., for pure cinnamaldehyde, a transparent layer was evident at the top of the nanoemulsion samples, showing that storage prompted the oil droplets to sink to the bottom of the test tube, and might be attributed to both the physical and chemical effects, such as chemical interaction, coalescence, Ostwald ripening, and sedimentation. In detail, cinnamaldehyde displays relatively high water-solubility, inducing droplet expansion via Ostwald ripening. Consequently, the mean particle size of the nanoemulsions expands, resulting in accelerated droplet coalescence and sedimentation [65].

## 4. Nanoemulsion Stabilizer

In order to satisfy the specific requirements of commercial applications, nanoemulsions should be designed to improve their kinetic stability, which is achieved through meticulous structure and composition control. Particularly, it is critical to select adequate aqueous and oil phases, as well as the most suitable additives, such as emulsifier, weighting agent, texture modifier, and ripening inhibitor [6]. The stabilization mechanism usually refers to the physicochemical properties of nanoemulsion such as composition, interfacial composition, electric charge, droplet size, physical state, aggregation state, rheology property and so on [3,6].

For instance, apart from decreasing the droplet size, gravitational separation can also be reduced by adding thickeners to improve the viscosity of the aqueous phase, or adding weighting agents to decrease the density contrast. Droplet aggregation due to flocculation and coalescence can be restricted by making sure that the repellent interactions (e.g., steric and electrostatic) of droplets exceed their attractive interactions (e.g., hydrophobic, van der Waals, and depletion). This is often accomplished by changing the aqueous phase composition or the nature of the emulsifier used [6]. The addition of ripening inhibitors or the utilization of an oil phase displaying low water-solubility, can restrict Ostwald ripening [6,77]. 

### 4.1. Emulsifier

During homogenization, the emulsifiers denoting a group of surface-active amphiphilic molecules adsorb onto the oil–water interface. Their primary function is to reduce the interfacial pressure to disrupt the droplets and create a defensive interfacial layer to restrict droplet accumulation [78]. Various emulsifier types are used in different industries for the stabilization and formation of nanoemulsions [79]. In the food industry, low-molecular-weight surfactant (LMWS, e.g., Tween series, Span series, phospholipid, glycolipid and so on), high-molecular-weight emulsifier (HMWE, e.g., protein and polysaccharide), and the mixture of them are commonly used [78]. The specific emulsifier that is used primarily ascertains the interfacial properties of nanoemulsions obtained, such as hydrophobicity, thickness, chemical reactivity, and electric charge. As a consequence, the choice of a suitable emulsifier is extremely important to prepare nanoemulsions for special applications.

#### 4.1.1. Low-Molecular-Weight Surfactant (LMWS)

LMWS usually consist of a defined hydrophilic head (which can be nonionic or charged) and a hydrophobic tail that is generally constituted of one or more acyl chains, may be synthetic or natural. The molecular weight of those LWMSs is between approximately 250 g/mol to approximately 1200 g/mol [78,79]. In this section, different kinds of LMWSs for nanoemulsions’ preparation are summarized in Table 3 and introduced as follows.

##### Synthetic Low-Molecular-Weight Surfactant (LMWS)

Examples of synthetic food-grade LMWS include mono- and diglycerides, sucrose esters, derivatives of monoglycerides, and polyoxyethylene derivatives, such as Tween series, Span series, sucrose monopalmitate and so on.

Li et al., prepared nanoemulsions using various surfactants and co-surfactants. Five suitable surfactant mixtures of surfactants were identified, including Cremophor EL/glycerol (1:1, m/m), Cremophor EL/1,2-propanediol (1:1, m/m), Tween 80/polyethylene glycol-400 (3:2, m/m), Tween 80/ethanol (3:2, m/m), and Tween 80/1,2-propanediol (3:1, m/m). All five nanoemulsions were exceedingly stable, exhibiting mean droplet sizes under 20 nm for a minimum of 28 days [80]. According to Rajitha et al., the preparation of nanoemulsions was achieved by using Tween 80 as surfactants. The prepared nanoemulsions displayed a mean particle size of 34 nm, a negative surface charge and a pH level that was skin compatible. The nanoemulsions were stable during storage in a refrigerator for the entire experimental period (3 months) [81]. According to Rebolleda et al., nanoemulsions containing particle sizes of 40 nm can be obtained by combining 1% oil with 7.3% surfactant mixture (37.4% of Span 80 and 62.6% of Tween 80). Nanoemulsions exhibited good stability when stored at 4 °C for 60 days and only a slight destabilization occurred in the last days of the storage at 25 °C for 60 days [82]. According to Llinares et al., nanoemulsions formulated by microfluidization with different HLB values (obtained by using different Tween 80 and Span 80 mixtures) and surfactant/ oil ratio. Stable nanoemulsions with lowest droplet size (2.88 nm) were obtained when HLB was 10.5 and surfactant/ oil ratio was 1 [50].

##### Natural Low-Molecular-Weight Surfactant (LMWS)

Although synthetic LMWS are extensively employed in the food industry, there remains increasing interest in natural, biobased alternatives because of their low cytotoxicity [78,94]. Phospholipids, glycolipids and saponins are usually used as natural LWMSs to produce nanoemulsions.

**Phospholipids.** Phospholipids are amphiphilic molecules naturally found in the cell membranes of animals, plants, and microorganisms, which are extensively used as emulsifiers in food products. The most common phospholipids in food-grade lecithins include phosphotidyletanolamine (PE), phosphatidylcholine (PC), phosphatidic acid (PA), and phosphatidylinositol (PI) [94]. 

According to Komaiko et al., nanoemulsions (d < 150 nm) can be formed from sunflower phospholipids. They have a surfactant-to-oil ratio higher that 1:1, as well as high PC levels produced the smallest droplets. Furthermore, electrostatic repulsion was primarily credited for the physical stability of the nanoemulsions. Therefore, the droplet accumulation occurred at high ionic strengths (electrostatic screening) and low pH levels (low charge magnitude) [83]. Artiga-Artigas et al. prepared nanoemulsions by using different kinds of surfactants (Tween 20, sucrose monopalmitate (SMP), or lecithin). Nanoemulsions with 2.0% w/w lecithin remained stable during a storage period of almost 86 days, while those containing Tween 20 or sucrose monopalmitate at equal concentrations demonstrated destabilization after 5 days and 24 h, respectively [84]. Furthermore, modified phospholipids have also been used as emulsifiers, but nearly no improved stability of modified phospholipids-stabilized nanoemulsions has been achieved so far. As reported by Cabezas et al., the nanoemulsions were prepared by using three modified sunflower lecithins (deoiled, hydrolysed, and fractionation with absolute ethanol) as emulsifiers. The fine O/W nanoemulsions stabilized by 1% volume of hydrolyzed sunflower lecithin showed the similar physical stability with those stabilized by the mixture of hydrolyzed sunflower lecithin and non-hydrolyzed lecithins (0.5/0.5 or 0.75/0.25, m/m) [85]. Furthermore, O/W nanoemulsions prepared with lysophosphatidylcholine (enzymatically modified PC) were less stable than those prepared with nature PC, despite their higher water affinity [86].

**Glycolipids.** Glycolipids are surface-active compounds generated by a variety of microorganisms comprised of fatty acids connected to a carbohydrate moiety. Rhamnolipids, sophorolipid, trehalolipids, cellobiose lipids and mannosylerythritol lipids denote the most prevalent glycolipids [95,96]. Among them, rhamnolipids were most usually used to prepare nanoemulsions. According to Deepika et al., a rhamnolipid biosurfactant manufactured using the mangrove sediment bacterium, *Pseudomonas aeruginosa* KVD-HR42, reduced the surface tension from 65.23 to 30.14mN/m at a critical micelle concentration value of 100mg/L, and showed excellent emulsion-forming capabilities. Furthermore, severe NaCl concentrations, temperature, and pH levels did not adversely affect the stability of the biosurfactant [87]. According to Bai et al., rhamnolipids were also suitable for use during the formation of nanoemulsions with small droplets (surface-weighted mean diameter (d_32_) < 150 nm). Rhamnolipid-coated droplets remained stable and could accumulate at various pH levels (5–9), temperatures (20–90 °C), and salt concentrations (<100 mM NaCl). However, droplet accumulation was evident in conditions involving high ionic strengths (200–500 mM NaCl), as well as highly acidic (pH 2–4) environments [68]. 

**Saponins.** Saponins can be derived from various natural sources and denote a substantial category of surface-active molecules comprised of hydrophobic areas such as phenolic structures, as well as hydrophilic areas, including sugar groups [94]. 

*Quillaja* saponins (QS) is commonly used to prepare nanoemulsions. Sedaghat et al., suggested that as a natural surfactant, QS can produce smaller nanoemulsions compared to SMP and octyl modified starch (O-MS). Additionally, Nanoemulsions stabilized by QS were more resistant to stress conditions (e.g., acidic pH and salt) while the nanoemulsions prepared with SMP were highly unstable [88]. According to Zhang et al., QS was superior to modified starch (MS) during nanoemulsion preparation, with the smallest mean particle size of 69 nm, while the turbidity was signified by 102 nephelometric turbidity units at 0.05% of the dispersed phase [89]. The effectiveness of numerous natural emulsifiers at preparing O/W nanoemulsions were compared. The results showed that QS and WPI required a lower amount of emulsifier to more efficiently induce nanoemulsion formation exhibiting droplets that were smaller and finer than other two emulsifiers [90]. 

Additionally, saponins from other sources can also be used as emulsifiers. According to Zhu et al., nanoemulsions with particle (d < 200 nm) were stabilized by tea saponins displaying fairly low surfactant-to-oil ratios. The subsequent nanoemulsions remained stable in conditions marked by a variety of salt concentrations (≤200 mM NaCl), temperatures (30–90 °C), and pH levels (pH 3–8). Moreover, an excellent long-term stability was observed when storing the nanoemulsions at varying temperatures (5 °C, 37 °C, and 55 °C) [91]. According to Shu et al., Ginseng Saponins (GS) were capable of producing nanoemulsions (volume mean diameter (d_4,3_) ≈ 125 nm) by HPH method. The obtained nanoemulsions were stable without droplet coalescence in the case of thermal treatment (30–90 °C, 30 min), storage (15 days at 5, 25 and 40 °C) and a limited pH level range. However, nanoemulsions fabricated from GS were unstable when salt was present (>25 mM NaCl) and when exposed to acidic environments (pH 3–6). The chemical stability of nanoemulsions relied significantly on the storage temperature [70]. According to Taarji et al. a natural extract from argan oil press-cake containing saponins could be utilized for producing nanoemulsion with limited particle sizes and excellent physical stability compared to those obtained with Tween 20. However, these nanoemulsions obtained were highly sensitive to the addition of salt (≥25 mM) and extreme acidic pH levels (pH < 3) [92], leading to instability. According to Gundewadi et al., nanoemulsions were obtained by USH method with particle size of 37.7–57.6 nm when saponin extracted from the pericarp of *Sapindus mukorossi* (0.4%) was used as biosurfactant [93].

#### 4.1.2. High-Molecular-Weight Emulsifier (HMWE)

HMWEs comprises of different types of water-soluble molecules, mainly proteins and polysaccharides [97]. In this section, different kinds of HMWEs for nanoemulsions’ preparation are introduced as follows and summarized in Table 4.

##### Protein

Many food proteins, derived from plants, animals, insects, and microorganisms, are amphiphilic molecules able to adsorb to the surfaces of oil droplets and stabilizing them against agglomeration. Additionally, proteins tend to adsorb at the interface of oil and water, creating a viscoelastic film that protects emulsion droplets against physical destabilization, particularly coalescence [134,135].

**Animal protein.** Proteins from animal origin, particularly from milk (whey protein (WP), casein and β-lactoglobulin), have been widely used as emulsifiers but with differences in their emulsification properties. Hwang et al. prepared nanoemulsions stabilized by whey protein concentrate (WPC). Nanoemulsions with spherical forms and particle sizes between 190 nm and 210 nm were obtained [98]. As mentioned above, Bai et al. found that nanoemulsions with fine droplets are formed more efficiently in the presence of WPI and QS than those of the other two emulsifiers, and require considerably less emulsifier, while smaller droplets are produced [90]. Ozturk et al. prepared nanoemulsions using two kinds of natural biopolymers (WPI and GA). The results showed that WPI was superior to GA in utilizing low emulsifier concentrations to produce small droplets [99]. According to Zhang et al., O/W sodium caseinate (SC) stabilized nanoemulsions exhibited desirable physical stability when subjected to heat treatment (80 °C, 90 min) and ion strength (100–500 mmol/L). However, nanoemulsions were prone to accumulation when the pH levels were in close proximity to the isoelectric point of the casein (pH 4–5) [100]. According to Kumar et al., SC nanoemulsions remained stable in differing processing conditions such as ionic strength (0.1–1.0 M), temperature (63–121 °C), and pH (3–7) [101]. According to Ali et al., nanoemulsions containing 1 wt% of β-lactoglobulin and 5 wt% of Miglyol 812 (the oil displaying the lowest viscosity level) exhibited an exceedingly small droplet size (about 200 nm), as well as low PDI. Additionally, the nanoemulsions were the most stable over 30 days at least [102].

**Plant protein.** Plant proteins have gained increasing interest as sustainable alternatives for their animal-based counterparts to produce nanoemulsions in recent years [136]. Xu et al. prepared nanoemulsions by ultrahigh pressure homogenization using soy protein isolate (SPI), β-conglycinin (7S) or glycinin (11S) (two major proteins of SPI) as emulsifier. All nanoemulsions exhibited desirable stability when subjected to different ionic strengths (0–500 mM NaCl), pH levels (<4 or >7), temperatures (30–60 °C) and storing periods (0–45 days) [103]. Schoener et al. fabricated nanoemulsions with pea protein as the emulsifier and found that the characteristics denoting digestion and bioaccessibility in the nanoemulsions relied on carrier oil types (corn, fish and flaxseed oil) [104]. Primozic et al. studied the influence of the concentration of lentil protein isolate (LPI) on the rheological nature, formation, and stability of O/W nanoemulsions. They found that the average particle size for all nanoemulsions ranged from 161 to 357 nm, unchanged over 28 days [105]. According to Tabilo-Munizaga et al., stable lentil protein-based nanoemulsions were obtained by the HPH method (above 200 MPa, 2 passes) using 1:1 of emulsifier: oil ratio [106]. According to Primozic et al., LPI was physically modified by HPH and used as an emulsifier. Comparing to the unmodified LPI-stabilized nanoemulsions, the nanoemulsions stabilized by modified LPI demonstrated a decline in the particle size to below 200 nm, increase in their storage stability, less protein aggregation and more digestible [107]. 

**Mixed animal protein and plant protein.** Due to the inferior emulsifying qualities of the plant proteins, researchers have consistently attempted to develop emulsions by using a combination of plant proteins and animal proteins, especially SC and micellar casein (MC) [110]. According to Ji et al., achieving stability in O/W nanoemulsions with uniform droplets (~250 nm, PDI <0.2) required combining SC, SPI and HPH for their formation [108]. Liang et al. studied the influence of the concentration levels and types of globular proteins on the flow behavior and physical characteristics of nanoemulsions stabilized by MC-globular protein mixtures. The results showed the formation of nanoemulsions (<300 nm), while the globular protein sources and the mixed MC-globular protein ratios exhibited no significant effect on the nanoemulsification. The heat stability of the mixed protein-stabilized nanoemulsions can be ranked as MC-SPI > MC-pea protein concentrate (PPC) > MC-WPC at equal globular protein ratios [109]. Wang et al. created nanoemulsions which were stabilized with a mixture of zein and SC. The subsequent nanoemulsions exhibited a spherical morphology with a negative surface zeta-potential (from −28.5 mV to −35.8 mV), and a clearly defined diameter (approximately 109 nm−139 nm). Moreover, the entrapment efficiency reached 84.24% with 2% (*m*/*v*) SC/zein at a 1:1 mass ratio. This formulation further indicated the most limited size distribution leading to exceptional stability during an ambient storage (22 °C) period of up to 30 days, while retaining excellent redispersibility following freeze-drying or spray-drying [25]. Yerramilli et al. prepared mixed protein-stabilized O/W nanoemulsions with a mixture of SC and pea protein isolate (PPI) (1:1) and then compared them with those prepared with SC or PPI alone. The mixed protein-stabilized nanoemulsions remained stable displaying no changes in droplet size and creaming over the 6-month storage period, while SC-stabilized nanoemulsions exhibited depletion-induced destabilization and PPI-stabilized nanoemulsions displayed extensive aggregation and enhanced viscosity [110]. Nanoemulsions prepared by the mixture of SC and PPI (1:1) were stable without significant changes in the droplet size during the storage of eight weeks [111]. 

##### Polysaccharides

Numerous natural or chemically altered polysaccharides were used to form and stabilize emulsions because they contained polar as well as non-polar groups on one molecule, such as GA, pectin, modified starch (MS) and so on [94].

**GA.** Gum Arabic (GA) is one of the natural polysaccharide emulsifiers most widely used in food and beverage. GA consists of a hydrophobic polypeptide backbone covalently attached to numerous hydrophilic anionic polysaccharide chains consisting of arabinose, galactose, rhamnose and glucuronic acid [137]. Moradi et al. prepared GA-stabilized nanoemulsions. The prepared nanoemulsions with a zeta-potential of −13.5 mV to −47.8 mV and particle sizes between 10.01 nm and 171.2 nm revealed the dilatant rheological qualities, as well as adequate radical scavenging antioxidant activity [112]. Bai et al. compared the effect of three different polysaccharide emulsifiers (GA, Beet Pectin (BP), and corn fiber gum) on the formation and stability of O/W emulsions. The results demonstrated that GA and BP were more efficient than corn fiber gum, and required less emulsifier, while producing smaller droplets [113]. As mentioned above, Ozturk et al. prepared nanoemulsions using two kinds of natural biopolymers (WPI and GA) and found that WPI was superior to GA in utilizing low emulsifier concentrations to produce small droplets. However, at elevated temperatures and high ionic strength, the nanoemulsions stabilized by WPI displayed flocculation instability near the protein isoelectric point, while the GA-stabilized nanoemulsions remained stable [99]. 

**Pectin.** Pectin, a complex polysaccharide, includes three domains, namely rhamnogalacturonan-I homogalacturonans, and rhamnogalacturonan-II [138]. The degree of methoxylation (DM) of pectin varied with the variety, botanical origin, extraction conditions, and plant maturity. High methoxylated pectin (HMP) with a DM of 60–80% are generally representative of commercial pectin derived from apple pomace or citrus peel [139]. Pectin has been used as an emulsifier alone, such as sugar beet pectin (SBP) and ultra-high methoxylated pectin (UHMP), but is usually examined as pectin-protein complexes due to a lack of sufficient lipophilic moieties to adsorb the oil phase [140,141]. 

SBP contain more acetyl groups at the O-2 and O-3 positions within the galacturonic backbone, as well as more covalently bound proteins evident in the lateral chains, and higher phenolic ester content. The presence of these protein and phenolic groups signal its adsorption to the oil-water interfaces, while reducing the interfacial tension [142,143]. As reported by Bai et al., the smaller droplet diameter (310 nm) was obtained at 1% SBP, rather than that of 3% GA (740 nm) and 5% corn fiber gum (4800 nm) [113]. UHMP can be prepared by esterification of common HMP. According to Hua et al., UHMP (molecular weight (MW) of 15,000 g/mol and DM of 91.02%) nanoemulsions prepared by the EPI method at 20%–50% of solid-to-oil ratios had a 400 nm approximate small mean particle size with a 17–20% creaming index. Additionally, the UHMP nanoemulsions were stable during 56 days of storage, as well as a 14-day storage period at pH levels of 2–8. Moreover, thermal treatment at a temperature of 85 °C accelerated the flotation capacity of large droplets without fracturing them. The good emulsion property of UHMP can be attributed to the fact that high DM caused by sufficient hydrophobic groups (e.g., methyl) could improve the lipophilicity of UHMP to adsorb the oil phase, and reduce charge repulsions, which enhanced the pliability and pH stability of the interfacial pectin. Furthermore, the small MW of UHMP induced the disintegration and motion of UHMP in the aqueous environment [114]. 

**Plant mucilage.** Plant mucilages are hydrocolloids of vegetable origin, usually extracted from the seeds, exudates, fruits, leaves, and tubers of upper plants. According to Martin et al. and Lago et al., the mucilage derived from the leaves of pereskia aculeata miller (or *Ora-Pro-Nobis* (OPN) in Brazil) is mainly a polysaccharide rich in arabinogalactan and consists of arabinose, galactose, galacturonic acid, and rhamnose, and is related to proteins. When OPN mucilage was used as an emulsifier, nanoemulsion (116 ≤ d_32_ ≤ 171 nm) with increased density, polydispersity, and zeta-potential were formed when using higher OPN mucilage concentrations and lower soybean oil levels [115,116]. According to Wu et al., compared with two commercial emulsifiers-GA and citrus pectin, water-soluble yellow mustard mucilage (WSMM) exhibited the better emulsion stability and the higher surface activity. Additionally, WSMM also showed the highest zeta-potential and the best storage stability, thermostability and freeze-thaw stability among the three polysaccharides [117].

**Octenyl succinic anhydride-modified polysaccharide.** Octenyl succinic anhydride (OSA) is usually used to modify polysaccharides, such as starch, β-cyclodextrin (β-CD), Konjac glucomannan (KG), GA, dextrin and so on [144,145]. 

OSA-modified starch (OSA-MS) is an effective emulsifier used to form various O/W emulsions due to the content of its additional dual-function (hydrophobic and hydrophilic) groups. Sharif et al., prepared nanoemulsions by using OSA modified waxy maize starch as an emulsifier. A monomodal size distribution was evident in the nanoemulsions demonstrating a mean particle size under 200 nm and a zeta-potential exceeding −30 mV, suggesting that the dispersed oil droplets experienced a strong electrostatic repulsion. Furthermore, the nanoemulsions displayed stability and shear thinning towards coalescence during storage (4 weeks at 25 ± 2 °C) and prolonged bactericidal activities [118]. Sharif et al., prepared nanoemulsions using two OSA-MS (Purity Gum Ultra and Purity Gum 2000) as emulsifiers. The results showed that Purity Gum ultra-stabilized nanoemulsions was higher than Purity Gum 2000-stabilized nanoemulsions after storage for four weeks at 40 °C [119]. According to Li et al., lycopene nanoemulsions stabilized by OSA-MS were fabricated using HPH. Lycopene molecules tended to reside in the hydrophobic core of the O/W nanoemulsion droplets in the presence of low lycopene concentrations. However, with increased lycopene loading, the molecules extended into the O/W interface, reinforcing the lateral OSA molecule packing on the interfacial membranes, while reduced mean particle size enhanced nanoemulsion stability [120].

Recently, OSA-β-cyclodextrin (OSA-β-CD) and OSA-Konjac Glucomannan (OSA-KG) have also been successfully used to stabilize nanoemulsions. According to Cheng et al., the nanoemulsions prepared by OSA-β-CD with different degree of substitution (DS) had a smaller oil droplet size and better storage stability compared with those of the nanoemulsions fabricated using β-CD [121]. Li et al. prepared nanoemulsions stabilized by OSA-KG. The maximum emulsification yield exceeded 95%. Additionally, the droplet size and PDI of the OSA-KG nanoemulsion were below 5 nm and 0.5, respectively, after 30 d of storage [122].

Some natural emulsifiers are ineffective during the formation and stabilization of oil-in-water emulsions when used separately but become effective when used in combination with others. Different combinations can be adopted to improve emulsifier functionality [146].

##### Mixture of Protein and Polysaccharide

As reported by Li et al., when SC, GA and whey protein hydrolysate (WPH) were used together as emulsifiers, they were spontaneously-ordered adsorbed on the droplet surfaces. The oil droplets of nanoemulsions obtained were fine and stable. Additionally, the stability of nanoemulsions was enhanced with an increase of the GA ratio, when the concentration of GA reached 2.0 wt%, the nanoemulsion always maintained homogeneity after storage for 30 days [123]. Silva et al. prepared nanoemulsions stabilized by WPI and WPI-chitosan mixture. The results indicated that a one-month storage period, as well as pH levels equal to those found in the stomach, were conducive to the stability of both nanoemulsions, while phase separation and creaming occurred at intestinal pH levels [124]. As reported by Sharma et al., nanoemulsions were fabricated using SC (5%) and pectin (0.1%) as emulsifiers by the HSH method. Nanoemulsions remained stable in all common food-processing conditions, except at pH 3.0–5.0. During storage at at 25 °C, the particle size of nanoemulsions increased from 172.1 ± 4.39 nm (0 th day) to 415.3 ± 23.38 nm (20 th day). The stabilization mechanism can be attributed to steric repulsion instead of electrostatic repulsion. In brief, the pectin adsorbed on the surface of the SC particles, leading to the formation of a thick layer during nanoemulsification. When density and thickness of the pectin layers could adequately maintain the casein particles at a distance substantial enough to prevent agglomeration via van der Waals attraction, the stability of nanoemulsions was attained [125].

##### Conjugate of Protein and Polysaccharide

According to many reports, chemically modifying proteins via polysaccharide conjugation can maintain their molecular integrity and improve their solubility and emulsifying characteristics, particularly at low pH levels (e.g., isoelectric point) [147]. Therefore, significant possibilities exist for using conjugate of protein-polysaccharides as an alternative to protein alone to prepare nanoemulsions.

So far, various protein–polysaccharide conjugates have been formed by Maillard reaction and used to prepare nanoemulsions. Fan et al. prepared nanoemulsions, which were stabilized with WPI and WPI-dextran (5 kDa, 20 kDa and 70 kDa) conjugates, with mean particle sizes of 156.8, 156.0 and 155.6 nm, respectively. These values were substantially lower than those stabilized with WPI (165.6 nm). Furthermore, the pH stability of the nanoemulsions was exhibited a marked improvement following glycosylation, particularly when the pH level approached the isoelectric point of 5.0. No significant creaming or flocculation was evident in any of the nanoemulsions after a 30-day storage period at 25 °C and 50 °C [126]. As reported by Farshi et al., nanoemulsions with particle size of 75 nm were prepared using the USH method with WPI-Guar Gum (GG) as an emulsifier. The significantly improved physical stability of the nanoemulsion was observed when WPI-GG (GG content of 0.1%–0.2% wt) was used [127]. Sonu et al. prepared nanoemulsions with the USH technique employing WP–maltodextrin (MD) conjugates with different ratios as emulsifiers. The mean zeta-potential, droplet size, and poly dispersity index of nanoemulsions prepared with 5.0% oil and 9.0% WP–MD (1:2 w/w) conjugate were -19.64 ± 0.23 mV, 116.60 ± 5.30 nm, and 0.205 ± 0.02, respectively. Additionally, nanoemulsions were stable during different types of food processing procedures (e.g., heat treatments (63 °C/30 min of pasteurization, 80 °C/10 min of fore warming, 100 °C/10 min of boiling, and 121 °C/15 min of sterilization), pH levels (3.0–7.0), ionic strength (0.1–1.0 M)), and storage conditions (e.g., 15 days at 25 °C) [128]. Liu et al., indicated that the emulsifying properties of the ovalbumin-D-lactose conjugate were considerably higher than ovalbumin at pH 7.0. Additionally, the nanoemulsions prepared by ovalbumin-D-lactose conjugate showed good pH, thermal, and storage stabilities [129]. Gharehbeglou et al. comparatively studied WP-pectin conjugate with small molecule surfactants for preparing and stabilizing double nanoemulsions. The results indicated that the minimum particle size of Tween 80-stabilized double nanoemulsions and WPC-pectin conjugate-stabilized ones were 98 and 100 nm, respectively. Additionally, the double nanoemulsions that were prepared using WPC-pectin conjugate presented a zeta-potential below −30 mV; demonstrating enhanced stability during long-term storage [130]. 

##### Conjugate of Protein/Peptide and Polyphenol

It has already been established that protein/peptide–polyphenol conjugates, especially the covalent conjugates, improve the antioxidant activity of proteins. The oxidative stability of nanoemulsions stabilized by protein/peptide–polyphenol conjugate is always higher than those stabilized by protein/peptide alone [148].

As reported by Yi et al., lactalbumin–catechin conjugate was used as an emulsifier to produce nanoemulsions. The nanoemulsions that were stabilized with lactalbumin and lactalbumin-catechin conjugates exhibited average droplet diameters of 158.8 and 162.7 nm, respectively [131]. As reported by Wang et al., Zein hydrolysate (ZH)–tannic acid (TA) complex was used as an emulsifier to produce nanoemulsions with mean particle size of 120 nm. The nanoemulsions that were stabilized with a ZH–TA complex presented significant encapsulation efficacy, as well as exceptional physical stability. Furthermore, the nanoemulsions that were stabilized using a ZH–TA complex displayed higher oxidative stability with lower levels of lipid hydroperoxides and volatile hexanal than when employing ZH alone for stabilization [132]. According to Pan et al., the emulsifying capacity of the rice protein hydrolysates (RPH) could be significantly enhanced following covalent interaction with chlorogenic acid (CA) at 0.025%. The nanoemulsions stabilized by RPH-CA conjugate possessed a remarkable physical stability, as evidenced by the lowest changes in size (80 nm) and zeta-potential (3.34 mV) of the nanoemulsions during storage. Moreover, oxidative stability of RPH-CA stabilized nanoemulsions was high to successfully restrict lipid oxidative degradation during storage [133]. 

#### 4.1.3. Mixture of High-Molecular-Weight Emulsifier (HMWE) and Low-Molecular-Weight Surfactant (LMWS)

In this section, different kinds of mixture of HMWS and LMWE for nanoemulsions’ preparation are summarized in Table 5 and introduced as follows.

##### Mixture of Protein and Surfactant

Acccording to Dey et al., stable fish oil nanoemulsion stabilized with combination of three emulsifiers (Sesame Seed Protein Isolate (SSPI), Span 80, and Tween 20) was fabricated. The nanoemulsion prepared with a mixture of 0.5% (w/v) of SSPI, Span 80 and Tween 20 (1:1) exhibited a reduced droplet size of 89.68 ± 2.375 nm, while the shelf-life stability improved during storage for eight weeks [149]. Li et al. produced nanoemulsions by HPH mehod using the mixture of SPI and PC as emulsifier. The best stability of nanoemulsions was observed when the content of SPI and PC was 1.5% and 0.22%, respectively, the homogenization condition was 100 MPa/4 times. Additionally, the average particle size, TSI and emulsification yield of the nanoemulsions obtained was 217 nm, 3.02 and 93.4%, respectively [150]. As reported by Shen et al., nanoemulsions were prepared by six different emulsifiers, including WPI, Polymerized Whey Protein (PWP), WPI-lecithin mixture, PWP–lecithin mixture, lecithin and Tween 20. All nanoemulsions obtained had the droplet size of the 194-287 nm, the entrapment efficiency of astaxanthin of 90% and showed good physical and chemical stability during storage at 4 °C [151]. 

##### Mixture of Polysaccharide and Surfactant

According to Zhong et al., nanoemulsions stabilized with PC are inherently unstable and prone to phase separation when exposed to particular environmental stresses, such as moderate ionic strength and low pH levels. The preparation and stability of PC-stabilized nanoemulsions could be improved when OSA-MS was combinedly used as emulsifiers [152]. Hu et al. developed nanoemulsions using lecithin and GA as specific emulsifiers, and studied their antimicrobial capabilities. The results indicated that the nanoemulsions presenting a particle size of 103.6 ± 7.5 nm were produced during the homogenizing aqueous phase (0.5% of GA, 0.5% of lecithin, w/v), in the presence of the essential oil mixture (1.25%, w/v) and ethanol (as a co-surfactant) [153]. 

##### Mixture of Protein, Polysaccharide and Surfactant

Cheong et al. fabricated nanoemulsions that were stabilized using SC, Tween 20 and β-CD and employing the HPH method. The optimum emulsifier concentration and process parameters were determined to be 10% (w/w) and 28,000 psi/4 cycles, respectively. The nanoemulsions were obtained with particle size of 122.2 nm, PDI of 0.147, and zeta-potential of −46.6 mV. Additionally, the nanoemulsions showed good stability during storage ==for up to 6 weeks at 25 ± 2 °C [154].

### 4.2. Weighting Agent

The weighting agent are hydrophobic substances with higher density than water, which include ester gum, brominated vegetable oil, sucrose acetate isobutyrate, rosin gum, and more [78]. Weighting agents are commonly employed in the formation O/W nanoemulsions. Since water generally displays a higher density than both triglyceride and flavor oils, adding a weighting agent at suitable proportions can increase the density of these compounds until their levels match that of the aqueous phase, therefore, reducing the tendency for gravitational separation and inhibiting creaming [6,78]. 

As reported by Llinares et al. adding rosin gum to the dispersed phase of nanoemulsions as weighting agent led to smaller droplet size values (from 580 nm to 350 nm), while elevated span values accelerated the emulsion creaming destabilization [155]. 

### 4.3. Texture Modifiers

Texture modifiers are substances that are generally included in the continuous phase of emulsions to achieve modified rheological properties, including thickening agents and gelling agents. Thickening agents typically comprise of soluble polymers displaying extended structures, and can achieve higher solution viscosity since it can modify the fluid flow profile. Gelling agents are capable of generating chemical or physical cross-linking with its neighbors and imparts solid-like properties to a nanoemulsion solution. The improved stability of nanoemulsions by texture modifiers can be attributed that the inhibition of droplet movement and thereby the retardation of gravitational separation [6,78]. Water-soluble polysaccharides and proteins are frequently employed as texture modifiers, i.g. thickening agents or gelling agents [6]. 

Many natural and semisynthetic polysaccharides, such as alginates, pectin, chitosan, carboxymethyl cellulose and so on, are usually considered as ideal thickening agent and gelling agent. The gelation of alginate needs addition of divalent cations or reduction in pH of solution. The gelation of pectin occurs at acidic pH or in the presence of calcium or other reagents. The gelation of chitosan can be obtained by the concentration of aqueous solutions. The gelation of carboxymethyl cellulose occurs in the presence of polyvalent cation [156]. So far, these water-soluble polysaccharides have been used as thickening agents or gelling agents in nanoemulsions. According to Artiga-Artigas et al., nanoemulsions were prepared by microfluidization with Tween 20 and sodium alginate as emulsifier and thickening agent, respectively. The smallest particle size was evident in these nanoemulsions at 261 nm, while exhibiting monomodal distributions with a 0.25 polydispersity index. Additionally, the viscosity, zeta-potential, and whiteness index of nanoemulsions were 22.7 mPa.s, –37 mV, and 57.28, respectively [157]. However, Salvia-Trujillo et al. observed both positive and adverse effects on the nanoemulsion stability when sodium alginate was added to nanoemulsions. The positive effect was that the addition of sodium alginate to the nanoemulsions improved their stability against lipid oxidation. The negative effect denoted that droplet flocculation remained when sodium alginate concentrations were higher than 0.05% (w/w) due to the appreciably increased viscosity of the nanoemulsions [158]. Guerra-Rosas et al. prepared nanoemulsions using Tween 80 and HMP as emulsifier and thickening agent, respectively. The smallest droplet size (11 ± 1 nm) was revealed in the HMP-thickened nanoemulsion [159]. According to Bai et al., polysaccharides (SBP, corn fiber gum) were used as thickening agents of Tween 80-stabilized nanoemulsions. The polysaccharide influence at an increasing solution viscosity and reducing flocculation, declined in the following order: SBP > corn fiber gum [66]. As reported by Thomas et al., nanoemulsion gels were created by incorporating nanoemulsions into 2 % chitosan. The permeation rate of the nanoemulsion gel (37 ± 0.5 °C) emerged at 68.88 μg/cm^2^/h, which was significantly lower than the value exhibited by the nanoemulsion (76.05 μg/cm^2^/h), indicating that the rat skin permeation in the nanoemulsion gel was restricted. However, the retention of curcumin on rat skin by nanoemulsion gel (980.75 ± 88 μg) is significantly higher than nanoemulsion (771.25 ± 67 μg) [160]. Arancibia et al. examined two thickeners, namely carboxymethyl cellulose (CMC) and starch, as well as the influence they had on the physical qualities and lipid bioavailability of based nanoemulsions. The results showed that starch-thickened nanoemulsions showed a smaller particle size (75.86–78.81 nm) and zeta-potential values (−1.3 to −6.9 mV) rather than that of CMC-thickened nanoemulsions (77.62–98.48 nm, −49.7 to −53.3 mV). Additionally, contrary to samples thickened with starch, the nanoemulsions that were thickened with CMC displayed enhanced stability and a lower release rate of free fatty acids following lipolysis [64]. 

Proteins are also used as thickening agent and gelling agent. Primozic et al. reported that the particle size distribution of flowable nanoemulsions prepared using LPI at 1–2 wt%, approached a larger peak following a storage period of 28 days. However, the size distribution in the gelled nanoemulsions that were prepared using 5 wt% LPI exhibited no changes from the original state since it remained a robust gel following a storage period of 28 days [105]. 

### 4.4. Ripening Inhibitor

The ripening inhibitors are substances that are added to the nanoemulsion dispersed phase to inhibit droplet expansion resulting from Ostwald ripening, therefore, disrupting the mixing effect [78]. These highly hydrophobic molecules exhibit and exceedingly low level of water solubility and are represented by long-chain triglycerides such as sunflower oil, grape seed oil, corn oil, palm oil and more. Generally, ripening inhibitors are employed in the preparation of O/W nanoemulsions containing highly water-soluble oil phases, including flavor and essential oils [6]. 

Ryu et al. studied the effect of ripening inhibitor types (corn oil, palm oil, coconut oil, and canola oil) on thyme oil emulsion stability, formation, and antimicrobial activity. A sufficient concentration of ripening inhibitor was determined to be around 40% of the oil phase, while continued antimicrobial activity during storage was evident in stable nanoemulsions with small oil droplets (d < 70 nm). The antimicrobial activity displayed by the nanoemulsions was determined by the specific characteristics of the ripening inhibitors used and their capacity to redirect hydrophobic antimicrobial components to the segregated hydrophobic domain, leading to a decrease in the following order: palm oil ≈ corn oil > canola oil > coconut oil [72]. As reported by Chang et al., the nanoemulsions prepared by combining an appropriate amount of ripening inhibitor (≥60 % corn oil or ≥50 % medium chain triglyceride (MCT)) with essential oil demonstrated physical stability [161]. As reported by Zhang et al., the addition of ester gum (EG) into the oil phase not only altered the viscosity of the phase, but also impeded Ostwald ripening. As a result, QS-stabilized nanoemulsions retained their stability during a storage period of two weeks at 23 °C, while nanoemulsions that were stabilized using MS displayed significantly higher turbidity and mean particle sizes [89].

The weighting agent, texture modifier and ripening inhibitor used for nanoemulsions’ preparation are summarized in Table 6.

## 5. Applications in Encapsulation of Bioactive Compounds

The use of many bioactive compounds, such as bioactive lipids, essential oils, flavor compounds, vitamins, polyphenols, carotenoids and so on in the food industry remain challenging due to their inadequate solubility in water as well as their stability in food preparations [14,162]. Generally, nanoemulsions are usually designed to retain bioactive compounds during storage within a food product but control their release when they encounter specific environmental conditions, such as the mouth for flavors or the gastrointestinal tract for pharmaceuticals or nutraceuticals [163]. Additionally, the delivery and slow release of hydrophobic bioactive compounds in O/W conventional emulsions or nanoemulsions are beneficial to enhance their bioaccessibility by improving their solubility and the incorporation of them into mixed micelles of the simulated gastrointestinal tract (GIT) system [164,165].

### 5.1. Bioactive Lipids

Essential polyunsaturated fatty acids (PUFAs), especially omega-3 oils, e.g., eicosapentaenoic acid (EPA), docosahexaenoic acid (DHA), α-linoleic acid and α-linolenic acid, are the main bioactive lipids [166,167]. PUFAs reportedly have substantial health benefits, such as the neuroplasticity of nerve membranes, nervous system activity, memory-related learning, cognitive development, synaptic transmission, and synaptogenesis [168]. However, bioactive lipids are highly unstable against oxidation and presented low odor thresholds. Therefore, exceedingly low concentrations affected the sensory parameters. Encapsulation of bioactive lipids in nanoemulsions is beneficial in reducing autoxidation, displaying compatibility with various food products, enhancing functional properties, solubilizing flavor or volatile compounds in lipids, while masking bitter or astringent tastes [169]. 

According to Zhang et al., stable DHA and EPA nanoemulsions were prepared by EPI method. Within 20 days, the best nanoemulsions have good physical stability under different storage conditions, and the retention rate of DHA/EPA can be stabilized at >60% [26]. Dey et al., suggested that nanoemulsions demonstrated a considerably higher uptake rate of PUFAs in three small intestinal regions, compared to conventional emulsions. Meanwhile, nanoemulsions showed a stronger resistance to lipopolysaccharide-induced nitric oxide production in the peripheral blood mononuclear cells of rats [49]. As reported by Karthik et al., DHA O/W nanoemulsions were fabricated by microfluidization using Tween 40, SC or SL as emulsifiers. Among them, Tween 40 stabilized nanoemulsions showed improved stability and extent of lipid digestibility [51]. 

### 5.2. Essential Oils and Flavor Compounds

Essential oils, in the form of aromatic volatile liquids, as well as semi-liquids, are commonly derived from the seeds, flowers, leaves, buds, fruits, bark, resins, and roots of plants. The antimicrobial activity provided by essential oils is beneficial against several types of fungi, Gram-positive bacteria, and Gram-negative bacteria [170]. The nature of essential oils, including hydrophobic, volatile and reactive, limits the incorporation of them into food matrices directly [171]. The significant application challenges regarding the incorporation of essential oils into food could be negated by their encapsulation in nanoemulsions. Essential oils loaded within nanoemulsions can be formed by two methods.

One method is homogenizing essential oils in the aqueous solutions containing emlsifier. According to Xue et al. and Ma et al., nanoemulsions containing essential oils (e.g., thyme oil, eugenol) were fabricated by directly homogenizing the essential oils in the aqueous solutions containing CS and lecithin, indicating that the antimicrobial characteristics were similar or exceeded those of the free essential oils [172,173]. As reported by Wang et al., eugenol (2-methoxy-4-(2-propenyl)-phenol), which forms a major part of clove essential oil, presents a wide range of antibacterial and antifungal activity. Eugenol-loaded nanoemulsions stabilized by the mixture of zein and SC were prepared by SE method. The results showed that the entrapment efficiency of eugenol was 84.24% when 1% (*v*/*v*) of eugenol and 2% (*m*/*v*) of SC/zein (1:1 mass ratio) were used. The obtained nanoemulsions demonstrated the most restricted size distribution, and retained remarkable stability throughout a storage period at ambient temperatures (22 °C, 30 days), while exhibiting exceptional redispersibility following freeze-drying or spray-drying [25]. According to Sharma et al., a synergistic O/W nanoemulsion containing clove and lemongrass oil was developed and its potential as antifungal agents was explored. The antifungal activity of clove and lemongrass oil was enhanced by formulation in nanoemulsions [174]. According to Moghimi et al., stable nanoemulsion of *Thymus daenensis* oil was produced by utilizing lecithin and Tween 80 as emulsifiers, with droplet size of 171.88 ± 1.57 nm. The nanoemulsions showed stronger antibacterial activity than pure oil against *Acinetobacter baumannii*, which is notoriously resistant to multiple drugs. This result is evidenced by the minimum inhibitory concentration of nanoemulsion and pure oil being 30–45 μg/mL and 62.5–87.5 μg/mL, respectively. Additionally, after incubation for 24 h, the nanoemulsions exhibited remarkable anti-biofilm activity at a sub-lethal dose (56.43% inhibition in 1/2 minimum inhibitory concentration) [55]. 

Another method is to pre-dissolve essential oils in commonly used oils and then emulsified in the aqueous phase. As reported by Yang et al., nanoemulsions containing the mixtures of citrus oils (e.g., bergamot oil and sweet orange oil) and common triacylglycerol oils (e.g., corn oil and MCT oil) with different mixing ratios were produced. The subsequent results suggested that the stability of nanoemulsions that contain mixed oil significantly exceeded that of nanoemulsions containing pure citrus oil [175]. As reported by Tian et al., it was difficult using pure cinnamaldehyde as the oil phase to form stable nanoemulsions. Stable nanoemulsions containing cinnamaldehyde were obtained with the addition of MCT. Additionally, nanoemulsions containing cinnamaldehyde and MCT could provide an enhanced long-term inhibition on the bacterial growth of *Escherichia coli* compared with pure cinnamaldehyde [23].

Recently, essential oil nanoemulsion-containing films have been produced and used for active food packaging. However, this aspect has been extensively described elsewhere [10] and is not repeated in the present review.

### 5.3. Vitamins

As essential micronutrients, vitamins form a crucial part of human health. There are two types of vitamins: fat-soluble (lipophilic) and water-soluble (hydrophilic). Vitamins A, E, D, and K are grouped as the lipophilic vitamins, while vitamins B and C are hydrophilic.

Lipophilic vitamins are biologically sensitive material, displaying marginal chemical stability and water solubility [176]. For instance, Vitamins A, and Vitamins E are easily oxidized, particularly when exposed to light, heat, light, and metal ions. Furthermore, visible and fluorescent light possessed the ability to alter the vitamin K structure dramatically. Vitamin D is the only element that was not significantly affected by the processing and storage environments [177]. Nanoemulsions are usually fabricated to improve their chemical stability, solubility and oral bioavailability.

Park et al. examined the stability of O/W nanoemulsions containing retinol (vitamin A) when exposed to ultraviolet (UV) light and subjected to a storage period at varying temperatures (4 °C, 25 °C, and 40 °C). The results suggested that UV light reduced the residual retinol in the emulsion systems that utilized low oil concentrations during preparation compared to bulk oil. However, oil concentrations exceeding 10 wt% caused residual retinol levels to be higher than in the bulk oil due to elevated emulsion turbidity [75]. According to Ji et al., the vitamin A palmitate retention ration in the nanoemulsion exceeded 93% following a storage period of three months at room temperature [108]. As reported by Kadappan et al., the nanoemulsions-based delivery system increased in vitro bioaccessibility of vitamin D_3_ by 3.94 folds, as evidenced by the significantly higher concentration of vitamin D_3_ in micelles. An animal study showed that the nanoemulsions significantly increased the serum 25(OH)D_3_ by 73% [178]. According to Schoener et al., the type of carrier oil (corn oil, fish oil and flaxseed oil) significantly affected the preparation, simulated gastrointestinal performance and stability of vitamins D_3_ nanoemulsions stabilized by pea protein. The results showed that the three lipids were all digested in the small intestinal simulation model within the first few minutes. Additionally, for the different carrier oils, both the digestion rate in simulated gastrointestinal and vitamin bioaccessibility declined in the following order: corn oil > fish oil ≈ flaxseed oil [104]. As reported by Lv et al., nanoemulsions containing vitamin E were fabricated by dual-channel microfluidizer, using corn oil as a carrier oil and using QS as an emulsifier. The optimized nanoemulsions resulted in a relatively high vitamin bioaccessibility (53.9%) [179]. According to Moradi et al., the cellular uptake of a-tocopherol in nanoemulsions displayed a rise of up to 12 times higher than microsized a-tocopherol [112]. As reported by Campani et al., nanoemulsions containing vitamin K_1_ have been prepared to overcome some difficulties associated to the incorporation of semi-solid vitamin K_1_ into food formulations. The results showed that nanoemulsions could offer an option for the commercial development of a liquid and aqueous formulation to deliver vitamin K_1_ [180].

### 5.4. Phenolic Compounds

Phenolic compounds displaying significant antioxidant properties can be employed in biological preparations and various food products such as anti-microbial, anti-atherogenic, anti-inflammatory, anti-thrombotic, and anti-allergenic agents [181]. Phenolic compounds are classified into lipophilic and hydrophilic compounds.

Lipophilic phenolic compounds were usually encapsulated by O/W nanoemulsions. O/W nano-emulsification, can reportedly improve the bioavailability of lipophilic phenolic compounds due to higher absorption, solubility, and permeation into the body, as well as the safeguarding of the lipophilic phenolic compounds in nanoemulsions within food preparations [169]. According to Kumar et al., curcumin nanoemulsions with sodium caseinate were prepared. The cellular uptake of curcumin was improved by nanoemulsification because that the slow release of curcumin in the intestine is beneficial to incorporate it into mixed micelles of the bile salts or phospholipids [101]. Zheng et al. prepared curcumin nanoemulsions by three different methods (e.g., pH-driven, conventional, and heat-driven) and compared them with three curcumin supplements that are currently widely available. The results showed that the bioaccessibility of all curcumin obtained nanoemulsions compared well to even the most superior commercial formulation. Additionally, the nanoemulsions produced using the pH-driven technique denoted the highest concentrations of curcumin in the mixed micelles phase following exposure to a simulation of a gastrointestinal tract [182]. Sugasini et al. prepared a phospholipid-stabilized nanoemulsion containing curcumin and carrier oil (sunflower oil, coconut oil, or linseed oil) and explored the possibility of nanoemulsions to enhancing the curcumin bioavailability and DHA levels in rats. The results indicated the presence of high DHA levels in tissue and serum lipids, as well as elevated curcumin levels in the serum, heart, liver, and brain of rats given feed nanoemulsions containing linseed oil and curcumin [183]. According to Silva et al., compared with WPI-nanoemulsions, nanoemulsions stabilized by WPI-chitosan mixture showed the improved apparent permeability coefficient of curcumin via Caco-2 cells, as well as the improved bioaccessibility and antioxidant ability [124]. According to Singh et al., the rate and extent of bioavailability of t-resveratrol was significantly enhanced by loading in nanoemulsions rather than that of free t-resveratrol. Alongside this, the results of an in situ single pass intestinal perfusion study showed a remarkable enhancement in the absorptivity and permeability parameters of nanoemulsions [184]. Son et al. prepared quercetin-loaded O/W nanoemulsions containing Tween 80, caprylic/capric triglyceride (Captex^®^ 355), soy lecithin, and sodium alginate using the SE method. The nanoemulsion polydispersity index and particle size were <0.47 and 207–289 nm, respectively. The nanoemulsions were stable at pH levels ranging from 6.5–9.0 during a storage period of three months at 21 °C and 37 °C. Additionally, in rats that received a diet high in cholesterol, the nanoemulsion containing quercetin displayed a more substantial efficacy in decreasing the level of serum and hepatic cholesterol, with higher release of bile acid into feces, compared to free quercetin [185]. As reported by Carli et al., nanoemulsion-encapsulated quercetin was created with the EIP method and using two separate surfactants, namely Brij 30, and Tween 80. Nanoemulsions were obtained with mean particle size of 180–200 nm. The retention of quercetin was around 70% in nanoemulsions that contained 0.30% quercetin (w/w) and were stored for 90 d. Additionally, the incorporation of quercetin-loaded nanoemulsions in chicken patés can improve their oxidative stability in a considerably more efficient manner than synthetic antioxidants. Sensory information suggested that the quercetin encapsulation in nanoemulsions enhances consumer acceptability of the products [186]. 

Hydrophilic phenolics or the mixture of hydrophilic and lipophilic phenolics were usually encapsulated by W/O nanoemulsions. According to Rabelo et al., stable W/O nanoemulsions containing açaí berry extracts (ABE, rich in anthocyanins) were successfully formulated. All W/O nanoemulsions containing different concentrations of ABE exhibited high antioxidant activity and retention rates of anthocyanins after 30 days of storage. When 2% of anthocyanins was encapsulated in a 30 wt% ϕd (weight fraction of the dispersed phase) W/O nanoemulsions, they had an estimated half-life of 385 days [187]. Moreover, hydrophilic phenolics can also be encapsulated by O/W nanoemulsions. As reported by Peng et al., The O/W tea polyphenols (TP) nanoemulsion were prepared with polysorbate 80 and corn oil using the HPH method. The TP nanoemulsions with particle sizes of 99.42 ± 1.25 nm were stable during a 20-day storage period at 4 °C, 25 °C, or 40 °C. The results of in vitro assay of the simulated digestion model displayed a higher degree of bioaccessibility with regard to (−)-epigallocatechin gallate (EGCG), while (−)-epicatechin (EC), (−)-epigallocatechin (EGC), and (−)-gallocatechin gallate (GCG) exhibited lower bioaccessibility in the nanoemulsions compared to the aqueous solutions [188].

### 5.5. Carotenoids

Carotenoids represent natural lipophilic pigments that provide various health advantages such as safeguarding the eyes and reducing certain cancers. Increasing carotenoid bioavailability can be achieved when they are ingested with dietary lipids since the micelles derived from digested products are beneficial to solubilization and transportation of carotenoids to the epithelial cells [169,189,190]. Encapsulation of hydrophobic carotenoids into O/W nnaoemulsions could protect them from external stress factors. Additionally, the bioavailability of carotenoids can be increased after nano-emulsification.

As reported by Fan et al., O/W nanoemulsions containing β-carotene (BC) were prepared using WPI and WPI-dextran as emulsifiers. Following a 30-day storage period at 25 °C and 50 °C, the highest BC retention rate was evident in nanoemulsions that were stabilized with WPI-DT (5 kDa) conjugate due to the relatively high scavenging ability of diphenyl-1-picryl-hydrazil (DPPH). Additionally, the encapsulation in nanoemulsions stabilized by WPI-dextran (70 kDa) significantly impeded the lipolysis and release of BC [126]. According to Yi et al., BC retention of lactalbumin-catechin conjugate-stabilized nanoemulsions was significantly greater than that of lactalbumin-stabilized ones, which was attributed to the increased radical-scavenging and binding ability with free metal ion of lactalbumin after grafting with catechin [131]. Meng et al. prepared nanoemulsions containing TP and BC and found that the addition of TP was effective in enhancing the oral bioavailability and storage stability of BC. During storage at varying temperatures of 4 °C, 25 °C, and 35 °C, the stability and the BC retention of nanoemulsions containing TP and BC was higher than those of nanoemulsions containing only BC. Additionally, as shown by the in vitro simulated digestion assay and the in vivo absorption study, comparing with nanoemulsions containing only BC, the nanoemulsions containing TP and BC exhibited the higher recovery rates of BC at digestion phases I and II and the higher conversion efficiency of BC to vitamin A [191]. As reported by Sotomayor-Gerding et al., carotenoid (astaxanthin or lycopene) nanoemulsions were obtained by the HPH method. Nanoemulsions were stable to environmental conditions and storage time. The nanoemulsion oxidative stability was improved by trolox and the stability of lycopene nanoemulsions was improved by the synergistic effect of trolox and butylated hydroxytoluene (BHT). Additionally, carotenoid nanoemulsions were partially (66%) digested and highly bioaccessible (70–93%) [192]. As reported by Liu et al., the bioaccessibility of astaxanthin in nanoemulsions containing different carrier oils (olive oil, flaxseed oil and corn oil) was much higher than that in nanoemulsions containing no lipid, due to that the hydrophobic carotenoids could be solubilized by the mixed micelles formed from the carrier oils. The final free fatty acid release, as well as the bioaccessibility of astaxanthin exhibited a decrease in the following order: olive oil > flaxseed oil > corn oil [193]. As reported by Shen et al., the nanoemulsions stabilized with WPI had the highest cellular uptake of astaxanthin, followed, in order, by PWP, WPI–lecithin mixture, PWP–lecithin mixture (5.05 ± 0.1%), lecithin, and Tween 20 [152].

## 6. Conclusions and Future Trends

Nanoemulsions have increased in popularity over the past few decades due to their specific characteristics, which include visible transparency, high surface area per unit volume, tunable rheology, and robust stability. 

The commonly used methods for preparing nanoemulsions include low-energy techniques (e.g., SE and PIT/PIC) and high-energy approaches (e.g., RSE, HPH, HPMF and USH). Nanoemulsions are thermo-dynamically unstable, and typically break down during storage due to a variety of physical mechanisms responsible for destabilization such as coalescence, flocculation, creaming, and Ostwald ripening. Furthermore, it is also possible for nanoemulsions to lose the characteristics responsible for their acceptability, or to break down over time due to various biochemical or chemical reactions, especially lipid oxidation. Therefore, nanoemulsions should be specifically designed with improved physical stability in mind (e.g., the addition of suitable stabilizers, including texture modifiers, emulsifiers, weighting agents, and ripening inhibitors) and chemical stability (e.g., by adding antioxidants). Researchers have used nanoemulsions extensively to encapsulate active constituents to improve their physicochemical stability and bioavailability. O/W nanoemulsions were usually used for encapsulation of bioactive lipids, essential oils, lipophilic vitamins, lipophilic phenolic compounds and carotenoid. 

Nevertheless, the concluding results and future trends arising from the recent findings are based on many published research papers. On the one hand, it is necessary to further study a wide range of bioactive compounds or food ingredients loaded in nanoemulsions to give a more comprehensive overview. On the other hand, most of the research studying the functionality of nanoemulsions are conducted in simple model solutions, while lacking research into the possible interactions between nanoemulsions and other macromolecules present in real food matrices, such as proteins, carbohydrates, fibers and so on. 

## Figures and Tables

**Table 1:** Examples of application of low-energy methods for nanoemulsions’ preparation.

Emulsification Method	Optimal Processing Conditions	Bioactive Compound Encapsulated	Droplet Diameter (nm)	Reference
SE	(1) titration of organic phase into aqueous phase, (2) constant stirring, 600 rpm, (3) room temperature	Peppermint essential oil	≈50	[20]
	(1) titration of organic phase into the aqueous phase, (2) constant stirring, 1000 rpm/10 min, (3) room temperature	Citrus oil	10–30	[21]
	(1) titration of organic phase into aqueous phase, (2) constant stirring, 750 rpm, (3) room temperature	Citrus oil	≈100	[22]
	(1) titration of organic phase into aqueous phase, (2) constant stirring, 600 rpm/15 min, (3) room temperature	Cinnamaldehyde	<100	[23]
	(1) stirred, 1000 rpm/1 h, (2) room temperature	Capsaicin	13–14	[24]
	(1) deprotonated eugenol in hot alkaline added to surfactant mixtures, (2) the mixtures were acidified to pH 7.0, stirred, 600 rpm	Eugenol	≈ 109–139	[25]
PIC	(1) mixed oil and surfactant, (2) oil phase added to aqueous phase, (3) phase inversion occurred at a certain oil-to water ratio, (4) stirred, 30 min	Docosahexaenoic acid Eicosapentaenoic acid	<200	[26]
	(1) aqueous phase (water, glycerol) added to organic phase (sunflower oil, polysorbate 80, curcumin), (2) stirred, 300 rpm/30 min	Curcumin	≈200	[27,28]
	(1) mixed organic phase and aqueous phase, (2) continuing stirred, (3) ambient temperature	Essential Oils Blend*	29.55–37.12	[29]
PIT	(1) all components were stirred, 30 min, (2) heated to 15 °C above the PIT, (3) the temperature was reduced to the PIT	Cinnamon oil	101	[30,31]
	(1) coarse emulsions were heated, 21–98 °C /0–3 h, (2) immediately quenching in ice/water with hand shaking	Lemon oil	≈100	[32]
	(1) mixing all components, (2) 3 temperature cycles (90–60–90–60–90–75 °C)	Curcuminoids	20–100	[33]

Note: essential oils blend* containing cape jasmine absolute, wan saw long oil, lemongrass oil and basil oil.

**Table 2:** Examples of application of high-energy methods for nanoemulsions’ preparation.

Emulsification Method	Optimal Processing Conditions	Bioactive Compound Encapsulated	Droplet Diameter (nm)	Reference
RSE	24000 rpm/25 min	docosahexaenoic acid	87	[46]
HPH	800 bar/8 cycles	docosahexaenoic acid	11.17	[46]
	103 M Pa/10 cycles	pepper extract	132 ± 2.0-145 ± 1.0	[47]
	60 MPa/3 cycles	curcumin	203.6-260.6	[48]
	40 kpsi/10 cycles	fish oil	89.7 ± 27.7	[49]
HPMH	137.9 MPa/10 cycles	rosemary essential oil	2.88	[50]
	1000 bar/5 cycles	docosahexaenoic acid	148	[51]
	350 bar/5 cycles	curcumin	275.5	[52]
	13 kpsi/1 cycle	fish oil	<160	[53]
USH	350 W/5 min	Resveratrol	20.41 ± 3.41	[54]
		resveratrol cyclodextrin inclusion complex	24.48 ± 5.70	
	20.5 kHz/400 W for 15 min	thymus daenensis oil	171.88 ± 1.57	[55]
Combined method	HPH (24,000 rpm/15 min) + HSP (800 bar/8 cycles)	docosahexaenoic acid	11.31	[46]

**Table 3:** Examples of low-molecular-weight surfactants (LMWS) for nanoemulsions’ preparation.

Types	LMWS	Reference
Synthetic LMWS	Mixture of Cremophor EL and glycerol/1,2-propanediol, Mixture of Tween 80 and PEG-40/ethanol/1,2-propanediol	[80]
	Tween 80	[81]
	Mixture of Tween 80 and Span 80	[50,82]
Natural LMWS	Sunflower Phospholipids	[83]
	Lecithin	[84]
	Modified Sunflower Lecithins (Deoiled, Hydrolysed, Fractionation with absolute ethanol)	[85]
	Lysophosphatidylcholine (Enzymatically Modified)	[86]
	Rhamnolipids	[68,87]
	QS	[88,89,90]
	Tea Saponins	[91]
	GS	[70]
	Argan Saponins	[92]
	Saponin extracted from the pericarp of *Sapindus mukorossi*	[93]

**Table 4:** Examples of high-molecular-weight emulsifier (HMWE) for nanoemulsions’ preparation.

Types	HMWS	Reference
Protein	WPC	[98]
	WPI	[90,99]
	SC	[100,101]
	β-Lactoglobulin	[102]
	SPI, 7S, 11S	[103]
	Pea Protein	[104]
	LPI	[105,106]
	LPI modified by HPH	[107]
	Mixture of SC and SPI	[108]
	Mixture of MC and Globular (SPI, PPC, WPC)	[109]
	Mixture of Zein and SC	[25]
	Mixture of SC and PPI	[110,111]
Polysaccharides	GA	[99,112,113]
	SBP	[113]
	UHMP	[114]
	Pereskia Aculeata Miller	[115,116]
	WSMM	[117]
	OSA-Starch	[118,119,120]
	OSA-β-CD	[121]
	OSA-KG	[122]
Mxiture of Protein and Polysaccharide	Mixture of SC, GA and WPH	[123]
	Mixture of WPI and Chitosan	[124]
	Mixture of SC and Pectin	[125]
Conjugate of Protein and Polysaccharide	Conjugate of WPI and Dextran	[126]
	Conjugate of WPI and GG	[127]
	Conjugate of WP and Maltodextrin	[128]
	Conjugate of Ovalbumin and D-lactose	[129]
	Conjugate of WPC and Pectin	[130]
Conjugate of Protein/peptide and Polyphenol	Conjugate of Lactalbumin and Catechin	[131]
	Conjugate of ZH and TA	[132]
	Conjugate of RPH and CA	[133]

**Table 5:** Examples of mixture of low-molecular-weight surfactant (LMWS) and high-molecular-weight emulsifier (HMWE) for nanoemulsions’ preparation.

Types	Mixture of LMWS and HMWE	Reference
Mixture of protein and surfactant	Mixture of SSPI, Span 80 and Tween 20	[149]
	Mixture of SPI and PC	[150]
	Mixture of WPI/PWP and Lecithin	[151]
Mixture of polysaccharide and surfactant	Mixture of OSA-Starch and PC	[152]
	Mixture of GA and Lecithin	[153]
Mixture of protein, polysaccharide and surfactant	Mixture of SC, β-CD and Tween 20	[154]

**Table 6:** Examples of weighting agent, texture modifier, ripening inhibitor for nanoemulsions’ preparation.

Types	Stabilizers	Reference
Weighting Agent	Rosin Gum	[155]
Texture Modifiers	SBP	[66]
	Corn Fiber Gum	[66]
	CMC, Starch	[64]
	Sodium Alginate	[157,158]
	HMP	[159]
	Chitosan	[160]
	LPI	[105]
Ripening Inhibitor	Corn Oil, Palm Oil, Coconut Oil, Canola Oil	[72]
	MCT	[161]
	Ester Gum	[89]

## Abbreviations

11S	Glycinin
7S	β-Conglycinin
β-CD	β-Cyclodextrin
ABE	Açai Berry Extracts
BC	β-Carotene
BHT	Butylated Hydroxytoluene
BP	Beet Pectin
CA	Chlorogenic Acid
CMC	Carboxymethyl Cellulose
CPI	Catastrophic Phase Inversion
DHA	Docosahexaenoic Acid
DM	Degree of Methoxylation
DPE	D-Phase Emulsification
DPPH	Diphenyl-1-Picryl-Hydrazil
DS	Degree of Substitution
EC	(−)-Epicatechin
EG	Ester Gum
EGC	(−)-Epigallocatechin
EGCG	(−)-Epigallocatechin Gallate
EIP	Emulsion Inversion Point
EPA	Eicosapentaenoic Acid
EPI	Emulsion Phase Inversion
ESD	Solvent Diffusion
GA	Gum Arabic
GCG	(−)-Gallocatechin Gallate
GG	Guar Gum
GS	Ginseng Saponins
HLB	Hydrophilic Lipophilic Balance
HPMH	High-Pressure Microfluidic Homogenization
HMP	High Methoxylated Pectin
HMWEs	High-Molecule-Weight Emulsifiers
HPH	High-Pressure Homogenization
HSH	High Speed Homogenization
KG	Konjac Glucomannan
LC-PUFAs	Long-Chain Polyunsaturated Fatty Acids
LMWSs	Low-Molecular-Weight Surfactants
LPI	Lentil Proteins Isolate
MC	Micellar Casein
MCT	Medium Chain Triglyceride
MD	Maltodextrin
MS	Modified Starch
MW	Molecular Weight
O/W	Oil-In-Water
O/W/O.	Oil-In-Water-In-Oil
O-MS	Octyl Modified Starch
OPN	Ora-Pro-Nobis
OSA	Octenyl Succinic Anhydride
OSA-KG	Octenyl Succinic Anhydride Modified Konjac Glucomannan
OSA-MS	Octenyl Succinic Anhydride Modified Starch
OSA-β-CD	Octenyl Succinic Anhydride Modified
PA	Phosphatidic Acid
PC	Phosphatidylcholine
PDI	Polydispersity Index
PE	Phosphotidyletanolamine
PI	Phosphatidylinositol
PIC	Phase Inversion Composition
PIT	Phase Inversion Temperature
PPC	Pea Protein Concentrate
PPI	Pea Protein Isolate
PWP	Polymerized Whey Protein
QS	Quillaja Saponins
RPH	Rice Protein Hydrolysates
RSE	Rotor-Stator Emulsification
SBP	Sugar Beet Pectin
SC	Sodium Caseinate
SE	Spontaneous Emulsion
SL	Soy Lecithin
SMP	Sucrose Monopalmitate
SPI	Soybean Protein Isolates
SSPI	Sesame Seed Protein Isolate
TA	Tannic Acid
TP	Tea Polyphenols
UHMP	Ultrahigh Methoxylated Pectin
USH	Ultrasonic Homogenization
W/O	Water-In-Oil
W/O/W	Water-In-Oil-In-Water
WI	Whiteness Index
WP	Whey Protein
WPC	Whey Protein Concentrate
WPH	Whey Protein Hydrolysate
WPI	Whey Protein Isolate
WSM	Water-Soluble Yellow Mustard Mucilage
